# *N*-[4-(β-d-Allopyranos­yloxy)benzyl­idene]methyl­amine

**DOI:** 10.1107/S1600536809000944

**Published:** 2009-01-10

**Authors:** Shi-Ming Lv, Lei Zheng, Hui Zhao, Ying Li, Shu-Fan Yin

**Affiliations:** a College of Chemistry, Sichuan University, Chengdu 610064, People’s Republic of China

## Abstract

The title compound, C_14_H_19_NO_6_, was synthesized by the condensation reaction between hecilid (4-formyl­phenl-β-d-allopyran­oside) and methyl­amine in methanol. In the crystal structure, the pyran ring adopts a chair conformation and adjacent mol­ecules are linked by inter­molecular O—H⋯O and O—H⋯N hydrogen bonds, forming a three-dimensional network.

## Related literature

For the pharmaceutical and biological properties of hecilid and its derivatives, see: Chen *et al.* (1981 ▶); Sha & Mao (1987 ▶); Zhu *et al.* (2006 ▶); Yang *et al.* (2008 ▶).
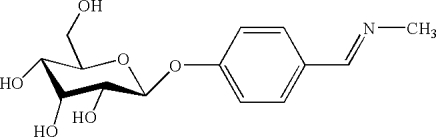


## Experimental

### 

#### Crystal data

C_14_H_19_NO_6_
*M* *_r_* = 297.30
Monoclinic, 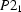

*a* = 6.721 (4) Å
*b* = 7.751 (3) Å
*c* = 14.119 (4) Å
β = 91.46 (3)°
*V* = 735.3 (6) Å^3^
*Z* = 2
Mo *K*α radiation
μ = 0.11 mm^−1^
*T* = 292 (2) K
0.48 × 0.46 × 0.44 mm

#### Data collection

Enraf–Nonius CAD-4 diffractometer
Absorption correction: none
1479 measured reflections
1469 independent reflections
1325 reflections with *I* > 2σ(*I*)
*R*_int_ = 0.004
3 standard reflections every 120 reflections intensity decay: 0.8%

#### Refinement

*R*[*F* ^2^ > 2σ(*F* ^2^)] = 0.034
*wR*(*F* ^2^) = 0.093
*S* = 1.09
1469 reflections
195 parameters
1 restraint
H-atom parameters constrained
Δρ_max_ = 0.16 e Å^−3^
Δρ_min_ = −0.25 e Å^−3^

### 

Data collection: *DIFRAC* (Gabe *et al.*, 1993 ▶); cell refinement: *DIFRAC*; data reduction: *NRCVAX* (Gabe *et al.*, 1989 ▶); program(s) used to solve structure: *SHELXS97* (Sheldrick, 2008 ▶); program(s) used to refine structure: *SHELXL97* (Sheldrick, 2008 ▶); molecular graphics: *ORTEP-3 for Windows* (Farrugia, 1997 ▶); software used to prepare material for publication: *SHELXL97*.

## Supplementary Material

Crystal structure: contains datablocks global, I. DOI: 10.1107/S1600536809000944/rz2284sup1.cif

Structure factors: contains datablocks I. DOI: 10.1107/S1600536809000944/rz2284Isup2.hkl

Additional supplementary materials:  crystallographic information; 3D view; checkCIF report

## Figures and Tables

**Table 1:** Hydrogen-bond geometry (Å, °)

*D*—H⋯*A*	*D*—H	H⋯*A*	*D*⋯*A*	*D*—H⋯*A*
O2—H2*O*⋯O5^i^	0.82	2.02	2.742 (3)	147
O3—H3*O*⋯O2^ii^	0.82	2.14	2.942 (3)	165
O4—H4*O*⋯O2^iii^	0.82	2.02	2.824 (3)	167
O5—H5*O*⋯N1^iv^	0.82	1.91	2.723 (3)	170

Symmetry codes: (i) 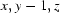; (ii) 
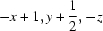
; (iii) 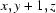; (iv) 
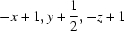
.

**Fig. 1.**
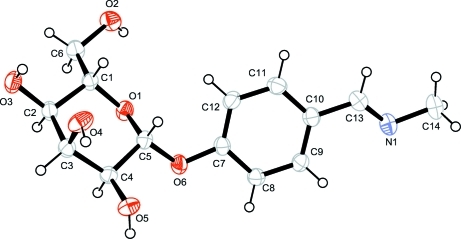
The molecular structure of the title compound, with displacement ellipsoids drawn at the 30% probability level.

## supplementary crystallographic information

### Comment

The natural compound hecilid (systematic name: 4-formylphenl-β-D-allopyranoside], which is extracted from the fruit of *Helicia nilagirica* Beed. (Chen *et al.*, 1981), has been one major active ingredient of herb medicine used in China for a long time. It has manifested good biological effects on the central nervous system and a low toxicity (Sha & Mao, 1987). Some derivatives of this compound have been reported with good pharmacological activities (Zhu *et al.*, 2006; Yang *et al.*, 2008). The title compound, a new helicid-derived compound, was synthesized *via* condensation reaction of hecilid and methyl amine with good yield.

In the molecule of the title compound (Fig. 1), the average of C–C bond length in the hexatomic ring is 1.524 (3) Å; The average C(*sp*^3^)–O and C(*sp*^2^)–O bond lengths are 1.421 (3) and 1.378 (3) Å, respectively. The hexatomic ring adopts chair conformation with the hydroxy group at C3 in axial position and the other substituents at C1, C2 and C4 in equatorial positions. The C(14)–N(1)–C(13)–C(10) and C(11)–C(10)–C(13)–N(1) torsion angles are -175.7 (3) and -165.5 (3) °, respectively, possibly as a consequence of O—H···.N hydrogen bond. In the crystal packing, intermolecular O—H···.O and O—H···.N hydrogen bonds (Table 1) link the molecules into a three-dimensional network.

### Experimental

A solution of helicid (1.42 g, 5 mmol) in methanol (8 ml) and a 40% aqueous solution of methyl amine (0.75 ml, 10 mmol) was subjected to ultrasonic radiation for 3 h at 333 K. On cooling to room temperature, colourless crystals were obtained unintentionally.

### Refinement

All H were positioned geometrically and refined using a riding model, with C—H = 0.93–0.98 Å, O—H = 0.82 Å, and with *U*_iso_(H) = 1.2*U*_eq_(C) or 1.5*U*_eq_(C, O) for methyl and hydroxy H atoms. In the absence of significant anomalous dispersion effects, Friedel pairs were averaged.

### Crystal data

**Table:**

C_14_H_19_NO_6_	*F*(000) = 316
*M**_r_* = 297.30	*D*_x_ = 1.343 Mg m^−^^3^
Monoclinic, *P*2_1_	Mo *K*α radiation, λ = 0.71073 Å
Hall symbol: P 2yb	Cell parameters from 25 reflections
*a* = 6.721 (4) Å	θ = 4.2–7.5°
*b* = 7.751 (3) Å	µ = 0.11 mm^−^^1^
*c* = 14.119 (4) Å	*T* = 292 K
β = 91.46 (3)°	Block, colourless
*V* = 735.3 (6) Å^3^	0.48 × 0.46 × 0.44 mm
*Z* = 2	

### Data collection

**Table:**

Enraf–Nonius CAD-4 diffractometer	*R*_int_ = 0.004
Radiation source: fine-focus sealed tube	θ_max_ = 25.5°, θ_min_ = 1.4°
graphite	*h* = −8→8
ω/2θ scans	*k* = 0→9
1479 measured reflections	*l* = −5→17
1469 independent reflections	3 standard reflections every 120 reflections
1325 reflections with *I* > 2σ(*I*)	intensity decay: 0.8%

### Refinement

**Table:**

Refinement on *F*^2^	Primary atom site location: structure-invariant direct methods
Least-squares matrix: full	Secondary atom site location: difference Fourier map
*R*[*F*^2^ > 2σ(*F*^2^)] = 0.034	Hydrogen site location: inferred from neighbouring sites
*wR*(*F*^2^) = 0.093	H-atom parameters constrained
*S* = 1.09	*w* = 1/[σ^2^(*F*_o_^2^) + (0.0607*P*)^2^ + 0.0722*P*] where *P* = (*F*_o_^2^ + 2*F*_c_^2^)/3
1469 reflections	(Δ/σ)_max_ < 0.001
195 parameters	Δρ_max_ = 0.16 e Å^−^^3^
1 restraint	Δρ_min_ = −0.24 e Å^−^^3^

### Special details

**Table:**

Geometry. All e.s.d.'s (except the e.s.d. in the dihedral angle between two l.s. planes) are estimated using the full covariance matrix. The cell e.s.d.'s are taken into account individually in the estimation of e.s.d.'s in distances, angles and torsion angles; correlations between e.s.d.'s in cell parameters are only used when they are defined by crystal symmetry. An approximate (isotropic) treatment of cell e.s.d.'s is used for estimating e.s.d.'s involving l.s. planes.
Refinement. Refinement of *F*^2^ against ALL reflections. The weighted *R*-factor *wR* and goodness of fit *S* are based on *F*^2^, conventional *R*-factors *R* are based on *F*, with *F* set to zero for negative *F*^2^. The threshold expression of *F*^2^ > 2σ(*F*^2^) is used only for calculating *R*-factors(gt) *etc*. and is not relevant to the choice of reflections for refinement. *R*-factors based on *F*^2^ are statistically about twice as large as those based on *F*, and *R*- factors based on ALL data will be even larger.

### Fractional atomic coordinates and isotropic or equivalent isotropic displacement parameters (Å^2^)

**Table:**

	*x*	*y*	*z*	*U*_iso_*/*U*_eq_	
O1	0.3071 (3)	−0.0753 (2)	0.21216 (11)	0.0379 (4)	
O2	0.4392 (3)	−0.3849 (2)	0.12078 (13)	0.0451 (5)	
H2O	0.4074	−0.4260	0.1717	0.068*	
O3	0.1787 (3)	0.0239 (3)	−0.03679 (11)	0.0441 (4)	
H3O	0.2854	0.0646	−0.0527	0.066*	
O4	0.3774 (3)	0.2704 (2)	0.06228 (14)	0.0465 (5)	
H4O	0.3892	0.3747	0.0704	0.070*	
O5	0.2525 (3)	0.3895 (2)	0.23888 (12)	0.0403 (4)	
H5O	0.1701	0.4234	0.2764	0.060*	
O6	0.3519 (2)	0.0903 (3)	0.34359 (10)	0.0394 (4)	
N1	1.0551 (3)	−0.0235 (3)	0.64875 (15)	0.0434 (5)	
C1	0.3201 (4)	−0.0893 (3)	0.11071 (17)	0.0331 (5)	
H1	0.4538	−0.0559	0.0915	0.040*	
C2	0.1662 (4)	0.0309 (3)	0.06374 (16)	0.0364 (5)	
H2	0.0333	−0.0081	0.0811	0.044*	
C3	0.1951 (4)	0.2142 (3)	0.09971 (16)	0.0377 (5)	
H3	0.0864	0.2873	0.0754	0.045*	
C4	0.2018 (4)	0.2204 (3)	0.20805 (16)	0.0336 (5)	
H4	0.0711	0.1891	0.2320	0.040*	
C5	0.3562 (4)	0.0917 (3)	0.24383 (15)	0.0336 (5)	
H5	0.4888	0.1242	0.2227	0.040*	
C6	0.2829 (4)	−0.2759 (3)	0.0851 (2)	0.0419 (6)	
H6A	0.1575	−0.3126	0.1110	0.050*	
H6B	0.2727	−0.2870	0.0167	0.050*	
C7	0.5264 (3)	0.0507 (4)	0.39210 (15)	0.0346 (5)	
C8	0.5361 (4)	0.1100 (4)	0.48498 (15)	0.0373 (6)	
H8	0.4298	0.1704	0.5098	0.045*	
C9	0.7042 (4)	0.0788 (4)	0.54001 (15)	0.0380 (6)	
H9	0.7102	0.1165	0.6026	0.046*	
C10	0.8654 (4)	−0.0086 (3)	0.50277 (16)	0.0372 (6)	
C11	0.8541 (4)	−0.0656 (4)	0.40913 (17)	0.0414 (6)	
H11	0.9618	−0.1226	0.3834	0.050*	
C12	0.6830 (4)	−0.0379 (4)	0.35401 (16)	0.0408 (6)	
H12	0.6742	−0.0788	0.2921	0.049*	
C13	1.0481 (4)	−0.0398 (4)	0.55985 (18)	0.0415 (6)	
H13	1.1629	−0.0727	0.5292	0.050*	
C14	1.2474 (4)	−0.0454 (5)	0.6982 (2)	0.0536 (7)	
H14A	1.3461	−0.0776	0.6535	0.080*	
H14B	1.2368	−0.1341	0.7453	0.080*	
H14C	1.2855	0.0611	0.7282	0.080*	

### Atomic displacement parameters (Å^2^)

**Table:**

	*U*^11^	*U*^22^	*U*^33^	*U*^12^	*U*^13^	*U*^23^
O1	0.0468 (9)	0.0314 (9)	0.0350 (9)	−0.0009 (8)	−0.0059 (7)	0.0028 (8)
O2	0.0549 (11)	0.0277 (9)	0.0526 (10)	0.0046 (8)	0.0014 (8)	0.0060 (8)
O3	0.0518 (11)	0.0434 (11)	0.0366 (8)	0.0007 (9)	−0.0127 (7)	−0.0042 (8)
O4	0.0700 (13)	0.0285 (9)	0.0416 (9)	−0.0075 (9)	0.0098 (8)	−0.0041 (8)
O5	0.0488 (11)	0.0329 (9)	0.0394 (9)	−0.0012 (8)	0.0074 (8)	−0.0061 (8)
O6	0.0371 (9)	0.0501 (11)	0.0307 (8)	0.0036 (8)	−0.0039 (7)	0.0020 (8)
N1	0.0370 (11)	0.0493 (14)	0.0435 (11)	−0.0017 (10)	−0.0059 (9)	0.0079 (10)
C1	0.0350 (12)	0.0281 (12)	0.0359 (11)	−0.0010 (10)	−0.0048 (9)	−0.0006 (10)
C2	0.0373 (13)	0.0342 (13)	0.0373 (12)	0.0019 (11)	−0.0098 (10)	−0.0010 (11)
C3	0.0453 (13)	0.0328 (12)	0.0347 (12)	0.0072 (12)	−0.0069 (10)	0.0012 (11)
C4	0.0365 (12)	0.0307 (12)	0.0335 (11)	0.0018 (11)	−0.0007 (9)	−0.0006 (10)
C5	0.0348 (12)	0.0368 (13)	0.0292 (11)	−0.0011 (11)	−0.0017 (9)	0.0012 (10)
C6	0.0464 (14)	0.0284 (13)	0.0504 (14)	−0.0019 (12)	−0.0091 (11)	−0.0016 (11)
C7	0.0364 (12)	0.0338 (12)	0.0334 (11)	−0.0003 (11)	−0.0033 (9)	0.0054 (10)
C8	0.0374 (13)	0.0401 (14)	0.0345 (12)	0.0043 (11)	0.0015 (10)	0.0000 (11)
C9	0.0410 (13)	0.0430 (14)	0.0297 (11)	0.0006 (11)	−0.0009 (9)	0.0004 (11)
C10	0.0381 (13)	0.0366 (13)	0.0368 (12)	−0.0006 (10)	−0.0021 (10)	0.0052 (10)
C11	0.0420 (14)	0.0412 (14)	0.0412 (13)	0.0085 (12)	0.0036 (10)	0.0018 (12)
C12	0.0491 (14)	0.0418 (14)	0.0315 (11)	0.0059 (13)	−0.0031 (10)	−0.0031 (11)
C13	0.0359 (13)	0.0411 (15)	0.0475 (14)	0.0003 (11)	−0.0003 (11)	0.0064 (12)
C14	0.0420 (15)	0.0617 (19)	0.0564 (16)	−0.0042 (15)	−0.0165 (12)	0.0100 (15)

### Geometric parameters (Å, °)

**Table:**

O1—C5	1.406 (3)	C4—C5	1.517 (3)
O1—C1	1.441 (3)	C4—H4	0.9800
O2—C6	1.430 (3)	C5—H5	0.9800
O2—H2O	0.8200	C6—H6A	0.9700
O3—C2	1.425 (3)	C6—H6B	0.9700
O3—H3O	0.8200	C7—C12	1.377 (4)
O4—C3	1.415 (3)	C7—C8	1.390 (3)
O4—H4O	0.8200	C8—C9	1.376 (3)
O5—C4	1.420 (3)	C8—H8	0.9300
O5—H5O	0.8200	C9—C10	1.392 (4)
O6—C7	1.378 (3)	C9—H9	0.9300
O6—C5	1.410 (3)	C10—C11	1.394 (3)
N1—C13	1.261 (3)	C10—C13	1.471 (4)
N1—C14	1.463 (3)	C11—C12	1.389 (4)
C1—C6	1.510 (3)	C11—H11	0.9300
C1—C2	1.531 (3)	C12—H12	0.9300
C1—H1	0.9800	C13—H13	0.9300
C2—C3	1.520 (4)	C14—H14A	0.9600
C2—H2	0.9800	C14—H14B	0.9600
C3—C4	1.530 (3)	C14—H14C	0.9600
C3—H3	0.9800		
			
C5—O1—C1	111.46 (17)	O6—C5—H5	110.4
C6—O2—H2O	109.5	C4—C5—H5	110.4
C2—O3—H3O	109.5	O2—C6—C1	111.5 (2)
C3—O4—H4O	109.5	O2—C6—H6A	109.3
C4—O5—H5O	109.5	C1—C6—H6A	109.3
C7—O6—C5	117.35 (18)	O2—C6—H6B	109.3
C13—N1—C14	118.3 (2)	C1—C6—H6B	109.3
O1—C1—C6	107.3 (2)	H6A—C6—H6B	108.0
O1—C1—C2	109.09 (19)	C12—C7—O6	124.5 (2)
C6—C1—C2	111.9 (2)	C12—C7—C8	121.0 (2)
O1—C1—H1	109.5	O6—C7—C8	114.5 (2)
C6—C1—H1	109.5	C9—C8—C7	119.5 (2)
C2—C1—H1	109.5	C9—C8—H8	120.2
O3—C2—C3	111.0 (2)	C7—C8—H8	120.2
O3—C2—C1	110.6 (2)	C8—C9—C10	120.5 (2)
C3—C2—C1	110.17 (18)	C8—C9—H9	119.7
O3—C2—H2	108.3	C10—C9—H9	119.7
C3—C2—H2	108.3	C9—C10—C11	119.2 (2)
C1—C2—H2	108.3	C9—C10—C13	121.3 (2)
O4—C3—C2	105.5 (2)	C11—C10—C13	119.5 (2)
O4—C3—C4	111.1 (2)	C12—C11—C10	120.5 (2)
C2—C3—C4	111.3 (2)	C12—C11—H11	119.8
O4—C3—H3	109.6	C10—C11—H11	119.8
C2—C3—H3	109.6	C7—C12—C11	119.2 (2)
C4—C3—H3	109.6	C7—C12—H12	120.4
O5—C4—C5	110.4 (2)	C11—C12—H12	120.4
O5—C4—C3	109.7 (2)	N1—C13—C10	122.6 (2)
C5—C4—C3	108.34 (19)	N1—C13—H13	118.7
O5—C4—H4	109.5	C10—C13—H13	118.7
C5—C4—H4	109.5	N1—C14—H14A	109.5
C3—C4—H4	109.5	N1—C14—H14B	109.5
O1—C5—O6	107.48 (19)	H14A—C14—H14B	109.5
O1—C5—C4	110.26 (19)	N1—C14—H14C	109.5
O6—C5—C4	107.84 (19)	H14A—C14—H14C	109.5
O1—C5—H5	110.4	H14B—C14—H14C	109.5
			
C5—O1—C1—C6	175.0 (2)	O5—C4—C5—O6	63.7 (2)
C5—O1—C1—C2	−63.6 (2)	C3—C4—C5—O6	−176.21 (19)
O1—C1—C2—O3	178.37 (19)	O1—C1—C6—O2	−66.7 (3)
C6—C1—C2—O3	−63.1 (3)	C2—C1—C6—O2	173.71 (19)
O1—C1—C2—C3	55.3 (3)	C5—O6—C7—C12	−21.4 (4)
C6—C1—C2—C3	173.8 (2)	C5—O6—C7—C8	157.8 (2)
O3—C2—C3—O4	−53.9 (2)	C12—C7—C8—C9	−0.4 (4)
C1—C2—C3—O4	68.9 (2)	O6—C7—C8—C9	−179.6 (2)
O3—C2—C3—C4	−174.57 (19)	C7—C8—C9—C10	1.2 (4)
C1—C2—C3—C4	−51.7 (3)	C8—C9—C10—C11	−0.5 (4)
O4—C3—C4—O5	55.8 (3)	C8—C9—C10—C13	178.6 (2)
C2—C3—C4—O5	173.1 (2)	C9—C10—C11—C12	−1.0 (4)
O4—C3—C4—C5	−64.8 (3)	C13—C10—C11—C12	179.9 (3)
C2—C3—C4—C5	52.6 (3)	O6—C7—C12—C11	178.0 (3)
C1—O1—C5—O6	−176.31 (17)	C8—C7—C12—C11	−1.1 (4)
C1—O1—C5—C4	66.4 (2)	C10—C11—C12—C7	1.8 (4)
C7—O6—C5—O1	90.4 (2)	C14—N1—C13—C10	−175.7 (3)
C7—O6—C5—C4	−150.8 (2)	C9—C10—C13—N1	15.4 (4)
O5—C4—C5—O1	−179.22 (19)	C11—C10—C13—N1	−165.5 (3)
C3—C4—C5—O1	−59.1 (2)		

### Hydrogen-bond geometry (Å, °)

**Table:**

*D*—H···*A*	*D*—H	H···*A*	*D*···*A*	*D*—H···*A*
O2—H2O···O5^i^	0.82	2.02	2.742 (3)	147
O3—H3O···O2^ii^	0.82	2.14	2.942 (3)	165
O4—H4O···O2^iii^	0.82	2.02	2.824 (3)	167
O5—H5O···N1^iv^	0.82	1.91	2.723 (3)	170

Symmetry codes: (i) *x*, *y*−1, *z*; (ii) −*x*+1, *y*+1/2, −*z*; (iii) *x*, *y*+1, *z*; (iv) −*x*+1, *y*+1/2, −*z*+1.
